# Editorial: Clinical Paths for Soluble Epoxide Hydrolase Inhibitors

**DOI:** 10.3389/fphar.2020.598858

**Published:** 2020-09-25

**Authors:** John D. Imig, Christophe Morisseau

**Affiliations:** ^1^ Cardiovascular Center, Medical College of Wisconsin, Milwaukee, WI, United States; ^2^ Department of Entomology and Nematology, University of California at Davis, Davis, CA, United States

**Keywords:** eicosanoids, oxylipins, heart disease, neurological diseases, kidney disease, pain, inflammation

## Introduction

Soluble epoxide hydrolase (sEH) is an enzyme that contributes importantly to metabolism of endogenous, biologically active arachidonic acid derived epoxyeicosatrienoic acids (EETs) (Imig and Hammock, 2009). Soluble epoxide hydrolase inhibitors (sEHIs) were developed as a means to increase lipid epoxides, including EETs. sEHIs were found to reduce blood pressure, improve insulin sensitivity, and decrease inflammation (Imig and Hammock, 2009). Further sEHI development led to initial clinical trials for hypertension and diabetes (Imig and Hammock, 2009). In recent years, there has been significant expansion of the potential clinical paths for sEHIs including clinical trials for COPD, with positive initial findings demonstrating improved endothelial function in smokers with COPD (Yang et al., 2017). There have also been great advances in sEHI development in the areas of chronic kidney disease, neuropathic pain, and metabolic diseases with clinical trials scheduled to begin for targeting diabetic neuropathic pain (Imig, 2018).

This Research Topic captures the increasingly broad scope of potential applications for sEHIs, covering cancer, ocular diseases, pulmonary, kidney, heart, liver, and neural pathologies. Research publications span preclinical animal studies, human studies, and the development of multi-target sEHI drugs. This Research Topic contains sixteen contributions that demonstrate the exciting clinical paths for sEHIs to treat human diseases.

## Mini Reviews and Reviews

Six review contributions to the Research Topic provide insight into the impact for sEH on physiological function and broad clinical potential for sEHIs. The influence of estrogen and sex on sEH and EET regulation and vascular function was the focus of a mini-review (Huang and Sun). The increased impact for sEHIs on cardiovascular performance and ischemic diseases for woman was compared to the potential for adverse impact on the pulmonary circulation (Huang and Sun). Another mini-review focused on the potential for sEHIs to treat ocular diseases (Park and Corson). This review covered eye diseases such as macular degeneration, retinopathy of prematurity, and diabetic retinopathy. A mini-review and review provided insight on psychiatric disorders and neurological diseases. These reviews expanded to include the epoxy fatty acids beyond EETs that sEHIs can regulate also. The therapeutic potential for sEHIs to regulate EETs and epoxydocosapentaenoic acids (EDPs) for psychiatric disorders was highlighted in a mini-review (Ren). Neural inflammation and sEH metabolism of polyunsaturated fatty acids (PUFAs) were the focus of a review (Hashimoto). This review covered sEHIs potential for treating depression and Parkinson’s disease (Hashimoto). A multi-target sEHI and cyclooxygenase (COX) inhibitor to enhance gastroenteropathy and inflammation-associated carcinogenesis was reviewed (Jones et al.). This review highlighted the ability for combined sEH and COX inhibition to impact mitochondrial function, reactive oxygen species, and inflammation (Jones et al.). Renal health in several kidney diseases and sEHIs was covered in a review (Liu). The potential for sEHIs to treat acute kidney injury, chronic kidney disease, hypertension induced kidney damage, and diabetic nephropathy were covered (Liu). These mini-reviews and reviews demonstrate the broad potential for sEHIs to treat diseases ranging from cardiovascular diseases, neurological diseases, kidney diseases, and ocular diseases.

## Drug Metabolism and Oxylipin Regulation

Drug and oxylipin metabolism are the topics in two scientific reports that are published in this Research Topic. The metabolism of an sEHI (TPPU) was investigated in rats and liver S-9 fractions from several species (Wan et al.). Findings demonstrated the ability to translate pharmacokinetic data for TPPU from rats to humans which facilitates clinical development of sEHIs (Wan et al.). Next, the effect of dimethylsulfoxide (DMSO) on oxylipin levels in mouse liver were investigated (Deol et al.). Data provided evidence that DMSO can decrease levels of oxylipin diols in mouse level and indicate caution when using DMSO as a vehicle for animal studies (Deol et al.). These studies highlight the predictability of rat studies for sEHI clinical development and that there are potential for vehicles in animal studies to influence oxylipin profiles.

## Heart Disease

The potential for sEHIs and EETs as a treatment for heart disease was the focus of three scientific studies published in this Research Topic. Lipopolysaccharide (LPS) myocardial inflammation and cardiotoxicity were studied in sEH null mice and in the presence of sEHI (Samokhvalov et al.). Accumulation of diols can contribute to LPS-induced cardiac cell mitochondrial dysfunction that can be combatted by sEHIs (Samokhvalov et al.). More chronic heart conditions can also be alleviated by sEHIs. The ability for sEHI to attenuate the progression of heart failure under conditions of chronic kidney disease were demonstrated in the Fawn-Hooded hypertensive rats (Vacková et al.). Another heart study evaluated the sEHI, c-AUCB, and EET analog, EET-A, to decrease postischemic heart failure in normotensive and hypertensive rats (Hrdlička et al.). Findings from this study indicate that sEHI or EET-based treatment attenuates the progression of post-myocardial infarction heart failure in normotensive but not hypertensive rats (Hrdlička et al.). Future studies are needed to translate these findings in animal studies for sEHIs and EET analogs to treat acute and chronic heart diseases in humans.

## Other Diseases

Three scientific articles in this Research Topic demonstrate the wide-ranging diseases that sEHIs and EET analogs could potentially treat. The first of these scientific articles evaluated sEHI to treat osteoarthritis a degenerative joint inflammatory disease (McReynolds et al.). In this study, 5 day treatment with the sEHI, EC1728, resulted in reduced inflammation and relieved pain in arthritic dogs (McReynolds et al.). The effect of sEHIs combined with docosahexaenoic acid to enhance the therapeutic ability in diabetic rats and neurological complications (Pardeshi et al.). This study demonstrates the beneficial anti-inflammatory and anti-oxidative actions of EDPs and sEHI on the brain to improve the memory response of diabetic rats (Pardeshi et al.). Lupus nephritis and treatment with the EET analog, EET-A, was the focus of the third scientific article (Hye Khan et al.). This study revealed decreased inflammation and fibrosis in lupus nephritis mice treated with EET-A that prevented progression of renal damage (Hye Khan et al.). These three scientific articles highlight the common threads of anti-inflammatory and anti-oxidative actions for sEHIs, the various avenues to manipulate epoxy fatty acids to achieve beneficial actions, and the breadth of human diseases that sEHIs and manipulating fatty acids have the potential to treat.

## Dual Inhibitors

One area where development of sEHIs has been expanding is as one component of multi-target drugs. The rational design of drugs that act on specific multiple targets has gained interest due to the recognition that the balanced modulation of two targets can provide a superior therapeutic effect and side effect profile. This balanced modulation is demonstrated in the scientific article demonstrating the effects of zafirlukast on 3T3-L1 adipocytes (Göbel et al.). Zafirlukast, which is a marketed CysLT1 receptor antagonist, provides the structural starting point for developing dual sEHI and proliferator-activated receptor γ activator (PPARγ) drugs that would have decrease inflammation, as well as, resolve inflammation to potentially treat chronic inflammatory diseases (Göbel et al.). The second multi-target sEHI is combined with COX-2 inhibition (PTUPB) and compared to a sEHI (*t*-TUCB) in an allergen-induced airway inflammation (Dileepan et al.). In this study the multi-target drug, PTUPB failed to provide additional advantage compared to *t*-TUCB; however, PTUPB could be useful in treating conditions where eosinophil and pain-associated inflammation co-exist (Dileepan et al.). These findings are two examples of the ever expanding multi-drug compounds with sEHI that are being developed. There is ample evidence that a multi-target drug with sEHI activity could eventually be successful in treating human diseases.

## Conclusions

The Research Topic Clinical Paths for Soluble Epoxide Hydrolase Inhibitors demonstrates the exciting potential for sEHIs to treat human diseases and improve quality of life. This collection of sixteen articles demonstrates that sEHIs can reduce inflammation, improve mitochondrial function, and decrease oxidative stress as mechanisms to combat disease. Therefore, there is great promise that an sEHI will be treating either ocular diseases, pulmonary diseases, kidney diseases, cardiovascular diseases, or neurological disorders in the near future.

## Author Contributions

JI: conceived the content and drafted the manuscript. JI and CM: revised and approved the final manuscript.

## Funding

This work was supported, in part, by the National Institute of Diabetes and Digestive and Kidney Diseases Grant DK103616, the National Institute of Environmental Health Sciences (NIEHS) Grant R35ES030443, NIEHS Superfund Research Program P42 ES004699, and the Dr. Ralph and Marian Falk Medical Research Trust Bank of America, N.A., Trustee.

## Conflict of Interest

JI has patents that cover the composition of matter for EET analogs and bifunctional sEH inhibitors. CM is an inventor on patents owned by UC Davis for composition of matter and usage of sEH inhibitors.
